# Biomechanical analysis of ocular diseases and its in vitro study methods

**DOI:** 10.1186/s12938-022-01019-1

**Published:** 2022-07-23

**Authors:** Yali Zhao, Guohuang Hu, Yuwei Yan, Zhen Wang, Xiaohua Liu, Huanhuan Shi

**Affiliations:** 1 The Fourth Hospital of Changsha, Affiliated Changsha Hospital of Hunan Normal University, Changasha, 410006 Hunan People’s Republic of China; 2 Department of Biomedical Engineering, School of Measuring and Optical Engineering, Nanchang Hangkong University, Nanchang, 330063 Jiangxi People’s Republic of China

**Keywords:** Ocular biomechanics, Ocular diseases, Finite element modeling, Microfluidic eye chip

## Abstract

Ocular diseases are closely related to the physiological changes in the eye sphere and its contents. Using biomechanical methods to explore the relationship between the structure and function of ocular tissue is beneficial to reveal the pathological processes. Studying the pathogenesis of various ocular diseases will be helpful for the diagnosis and treatment of ocular diseases. We provide a critical review of recent biomechanical analysis of ocular diseases including glaucoma, high myopia, and diabetes. And try to summarize the research about the biomechanical changes in ocular tissues (e.g., optic nerve head, sclera, cornea, etc.) associated with those diseases. The methods of ocular biomechanics research in vitro in recent years are also reviewed, including the measurement of biomechanics by ophthalmic equipment, finite element modeling, and biomechanical analysis methods. And the preparation and application of microfluidic eye chips that emerged in recent years were summarized. It provides new inspiration and opportunity for the pathogenesis of eye diseases and personalized and precise treatment.

## Introduction

The study of biomechanics can provide the mechanical forces information involved in biological processes from organs to cells level [1–3]. Each tissue inside and outside the eyeball has a certain tension, which is interrelated and affects each other in a closed spherical organ [4]. More and more attention has been paid to the study of the biomechanics of eye tissues to achieve the purpose of prevention and treatment of ocular diseases [5]. The pathogenesis of common and refractory eye disease has been studied through computational simulation modeling of eye tissue, to explore the biomechanical mechanism of ophthalmic diseases. It will lay a solid foundation for the development of modern ophthalmology precision medicine [6, 7].

At present, biomechanical studies on pathological ophthalmology-related diseases mainly focus on glaucoma [8], high myopia [9], and diabetic eye disease [10]. Biomechanics is an important part of the research on the pathogenesis, prevention, and treatment of glaucoma. High intraocular pressure (IOP) is the main risk factor for visual function injury. The increase of outflow resistance of the trabecular meshwork is the main factor in the increase of IOP. The increase of IOP will lead to iris deformation, pupil block, changes in the flow field of the anterior atrial, thinning of optic nerve fiber layer and lamina cribrosa (LC), visual function injury, etc. [11]. IOP is an important biomechanical factor directly related to glaucoma. Eye contents include aqueous humor, lens, and vitreous body, among which aqueous humor has the greatest influence on IOP. However, normal IOP can also lead to glaucoma, which is known as normal-tension glaucoma. In addition to the IOP, the biomechanical characteristics of the optic nerve head (ONH), LC, sclera, iris, pupillary block, aqueous humor outflow system, and trabecular meshwork associated with glaucoma also should be discussed [12, 13].

Moreover, the sclera is very important in the biomechanical study of myopia pathogenesis and treatment. Besides working with the cornea to maintain a refractive state, the sclera provides stable mechanical support to delicate ocular structures such as the retina and optic papilla. Diabetes causes many ocular complications, such as corneal epithelium, corneal stroma, corneal endothelium, which may result in further biomechanical changes in microstructure. Therefore, the biomechanical research progress related to the above diseases was summarized in the following text (Table 1).

**Table 1:** Biomechanical analysis of various ocular diseases

Ocular diseases category	Research contents of biomechanics	Problems	Refs
Glaucoma	Exploration of the pathogenesis of glaucoma, biomechanical changes of optic nerve head, sclera, cornea, etc.	The biomechanical changes obtained by the simplified model are not accurate enough	[15–21]
High myopia	Biomechanics of ocular tissue changes in high myopia and glaucoma, blindness, etc.	The cost of animal experiments is high, and the precision of in vitro model experiments is not high	[9, 22–29]
Diabetic eye diseases	Relationship between biomechanical changes in diabetic eye disease and glaucoma, blindness	Lack of accurate in vitro models	[30–37]

In vitro biomechanics research methods for ophthalmic diseases are mainly divided into medical equipment, finite element modeling, and the recently emerging microfluidic research [14]. Ophthalmic physiotherapy equipment like Corvis ST is mainly used to directly measure IOP or biomechanical parameters. The finite element modeling method is still focused on the study of glaucoma disease. Microfluidic-based eye chip research includes the preparation of eyeball drugs using on microreactor. The preparation of eye tissue chips through in vitro cell culture and microfluidic-based drug-release devices, etc., may provide a new idea for personalized eye disease research. Finite element modeling and in vitro eye organ chips may provide direction for the subsequent development and application of smart wearable contact lenses.

The main purpose of this review is to summarize the biomechanical research hotspots of common ophthalmic diseases in recent years, and to introduce the in vitro research methods around the biomechanics of these ocular diseases, hoping to provide reference for relevant researchers.

## Biomechanical analysis of ocular diseases

Biomechanics is a powerful tool for the study of ophthalmic diseases. This part summarizes the research progress of common ophthalmic diseases in recent years, including glaucoma, high myopia and diabetic ocular diseases, hoping to help researchers have an understanding of this research area and provide new ideas for future research.

### Glaucoma

The progressive death of retinal ganglion cells (RGC) in ONH is the main cause of glaucoma blindness. Poor aqueous humor drainage can lead to an increase in IOP, resulting in optic nerve papilla injury. Damage to the optic nerve caused by pathological structural changes can also lead to glaucoma even under normal IOP. This suggests that a full understanding of the biomechanical properties of the peripapillary sclera and the cornea may be helpful for the research and treatment of glaucoma [2].

It is necessary to develop a method for modeling individual differences in the optic nerve. Schwaner et al. [15] proposed a biomechanical modeling method of the rat optic nerve papilla for individual glaucoma studies. The three rat models of ONH are greater than that of human. The rat model of glaucoma ONH strain results were compared to learn more about the connection between the biomechanics of glaucoma and cell death. However, due to the model simplification, this method also has limitations. So, to refine the model, they [16] constructed and analyzed a model of the optic nerve head with an individual specific geometry, in which the sclera was modeled as a matrix reinforced by collagen fibers. The situation of elevated IOP was simulated. Through the data of rat glaucoma study, the mechanism of biomechanical influence on glaucoma retinal ganglion cell lesions in individual differences could be further explored. They also [17] established a parameterized model of the rat optic nerve for sensitivity studies. The results indicated that scleral properties had an important impact on the biomechanics of the rat optic nerve. Scleral thickness, scleral fiber arrangement, scleral fiber hardness, and scleral matrix hardness were the most influential parameters on the biomechanics of the optic nerve. So, for future modeling studies, specific values of these parameters should be determined to provide a more accurate research model of individual differentiation.

Moreover, glaucoma with normal intraocular pressure was also studied. Chen et al. [18] concluded that the optic nerve strain caused by axial extension is closely related to the unilateral normal-tension glaucoma pathological mechanism, similar to the cornea and sclera biomechanics. In patients with thin central cornea thickness, the correlation between central cornea thickness the strain rate of the optic nerve, and the strain rate of orbital fat were statistically significant [19]. Additionally, by measuring the response of the deformation of the sclera around the astrocyte layer and the adjacent papillary in mice to the increase of IOP, Korneva et al. [20] investigated the biomechanics of ONH and peripapillary sclera in a mouse model with glaucoma. The results showed that the mechanical behavior of the astrocyte layer and peripapillary sclera at the site of glaucoma neuron injury and remodeling changed dynamically over time.

Biomechanical mechanisms are thought to partly explain glaucoma optic neuropathy, Wei et al. [21] analyzed dynamic corneal response parameters by comparing normal-tension glaucoma subjects, hypertension glaucoma subjects, and control subjects. Results showed that the corneal deformation of normal-tension glaucoma was more pronounced than in hypertension glaucoma or the control group, people with hypertension glaucoma showed no significant difference in corneal deformation ability compared with the control group. The thinner the cornea, the lower the IOP, and the more easily the cornea deformation. These factors should be considered in the diagnosis of glaucoma.

### High myopia

With the increase of myopia, excessive axial extension after the eyeball shows the biomechanics of stretching, followed by a series of retinopathy can cause significant vision loss. These may result in temporary or permanent loss of vision. The biomechanical changes of the sclera and cornea caused by myopia are the focus of myopia biomechanical research. To study the volume change of collagen fiber bundle structure behind sclera in highly myopic human eyes, Markov et al. [22] used the wide-angle X-ray scattering method to locate collagen in the sclera of non-myopic and highly myopic eyes. In high myopia, the normal post-scleral collagen microstructure changes greatly. These changes may reflect remodeling of the posterior sclera during axial elongation and/or mechanical adaptation to tissue pressure caused by fluid pressure or eye movement. Progression of myopia is thought to be associated with weakened scleral biomechanics, which results in ocular tissue deformation and axial elongation.

To strengthen weakened sclera and control myopia, scleral cross-linking has been proposed. The biomechanics of scleral weakening and scleral strengthening after cross-linking in myopia is not entirely clear. For investigating the effect of lysine oxidase (LOX) alone or in combination with Genipin on sclera cross-linking in myopic eyes, Wang et al. [23] compared the effects of cross-linking LOX and Genipin on the biomechanics and fixation index of guinea pig sclera. LOX crosslinked sclera in normal and myopic eyes. The cross-linking effect of LOX was weaker than that of Genipin, and the catalytic cross-linking effect of LOX and Genipin was not found in this study. Levy et al. [24] used Genipin to inhibit the scleral cyclic softening in tree shrews to investigate the increase in experimental myopia and scleral cross-linking. The results revealed that the sclera of young tree shrews was inelastic and cyclically softened by the cyclic tensile load.

It is well known that myopia alters the scleral structure and biomechanical properties, but its effect on corneal biomechanics is not well known. To further evaluate the corneal of myopia, Kang et al. [25] used an optical coherence tomography (OCT)-indentation probe, and found that the corneas of chicks with high myopia were more curved and softer on all IOP tests. Han et al. [26] explored that when there is a higher spherical equivalent in different degrees of myopia, the corneal stress–strain index is lower. It is suggested that the corneal mechanical strength might be compromised for high myopia. In addition, Yu et al. [27] observed the biomechanics and height of corneal after small incision lenticule extraction (SMILE) and laser-assisted subepithelial keratoplasty (LASEK). The effect of SMILE on corneal biomechanics might be smaller than that of LASEK in early postoperative removal of unit corneal tissue, but is comparable in long-term observation. Liu et al. [28] found that femtosecond-assisted LASEK (FS-LASIK) had less effect on corneal biomechanics than LASIK when assuming the same central corneal thickness for high myopia. The risk of corneal dilation after LASEK is lower than that after FS-LASIK.

Furthermore, highly myopic eyes are at significantly increased risk of many different secondary diseases due to morphological and structural changes [29]. High myopia may result in the risk of retinal detachment after lens surgery. Understanding the associated risk profile is clinically important. Grytz et al. [9] found that both myopia and glaucoma are chronic diseases leading to the connective tissue remodeling of the sclera and ONH. The mechanobiology behind connective tissue remodeling between the two diseases is essentially different, with different homeostasis control mechanisms.

### Diabetic ocular disease

The blood glucose value increases in diabetes, which may result in a lot of eye diseases. It can affect the front and back segments of the eye and lead to severe vision defects and even blindness. The retina tissues could be acutely affected by diabetes, almost all ocular diseases may happen in diabetic patients [10]. The structure of the cornea and sclera will also be influenced by poor blood glucose control [7]. A series of studies will provide the possibility of simple and non-invasive treatment for diabetic patients, as well as may find new methods for early treatment of related complications.

Sahin et al. [30] measured IOP with an ocular response analyzer (ORA) and Goldman applanation tonometer (GAT) to explore the corneal biomechanical changes and their influence on IOP measurement in diabetic patients. The results showed that diabetes affected the corneal biomechanics and resulted in lower corneal hysteresis than the healthy controls, leading to clinical ocular hypertension. Kotecha et al. [31] used ORA to evaluate corneal hysteresis and corneal response in diabetic patients and nondiabetic patients, and explored that changes in corneal biomechanics in diabetic patients may be related to blood glucose concentration.

Furtherly, Scheler et al. [32] explored corneal hysteresis and corneal resistance factor in poorly controlled diabetes was more severe than those of healthy people and well-controlled diabetic subjects. It suggested that biomechanical properties of the cornea depending on glycemic control. Bao et al. [33] also found the mechanical stiffness of the cornea of diabetic rabbits was significantly increased, which was manifested by the increase in corneal thickness and tangent modulus. Ramm et al. [34] detected corneal hysteresis and found corneal resistance factor was significantly increased in diabetic patients by using ORA and Corvis ST. And then, Ramm et al. [35] collected and evaluated age, IOP, and central corneal thickness of diabetic patients and healthy people. They assessed the effect of disease-specific factors and established a reliable sensitivity and specificity for diabetes mellitus values.

Same findings as above, Beato et al. [36] compared the corneal biomechanics after phacoemulsification in diabetes people and those without diabetes. The recovery of corneal hysteresis in diabetes was slower than in nondiabetic patients, while both groups were subjected to a significant and continuous decrease of the IOP and corneal resistance factor in phacoemulsification. In addition, Terai et al. [37] found that ONH stiffness was notably increased in diabetic rats compared with the peripapillary sclera, which may be related to non-enzymatic collagen cross-linking mediated by late glycation end products induced by diabetic hyperglycemia. To make clear whether these biomechanical changes have adverse risk factors for IOP regulation in diabetic people with glaucoma, further studies are needed to do.

## In vitro biomechanical study methods of eye tissue

Noninvasive in vitro study can provide more accurate and a large amount data for ocular biomechanics analysis, this part summarizes the recent study about the biomechanical measurement equipment, the finite element modeling and microfluidic chip in vitro method for ocular disease study.

### Ocular biomechanical analysis using medical ophthalmic equipment

Measurement of ocular parameters with ophthalmic professional medical equipment is an important basis for clinical diagnosis and treatment [38]. In the study of human eye data, a clinical visual corneal biomechanics analyzer (Corvis ST) is mainly used. Glaucoma, for example, to assess the reaction parameters on the same day the dynamics of the cornea patients, springback tonometer measurement of IOP, corneal thickness, and ocular axial length. It is beneficial for the analysis of normal-tension glaucoma and unilateral normal-tension glaucoma pathogenic factors [39]. Hong et al. [40] reported a new biomechanical parameter corneal flattening speed by Corvis ST measurement to determine whether it is related to the diagnosis of glaucoma with normal IOP. The speed of corneal flattening inward in normal IOP glaucoma was smaller than that in normal IOP glaucoma, which was statistically significant.

Additionally, Pradhan et al. [41, 42] compared corneal biomechanics in pseudoexfoliation syndrome, sham stripping pseudoexfoliation glaucoma, primary open-angle glaucoma, and healthy controls with Corvis ST, and found the nearly the same results for those corneal biomechanical parameters. Vinciguerra et al. [43] compared Corvis ST and Goldmann tonometers in primary and normal open-angle glaucoma, high IOP, and control group. Moreover, they compared the dynamic corneal response parameters of Corvis ST for primary high IOP, the high IOP, and the control group, suggesting that corneal biomechanics may be an important factor affecting IOP measurement. Abnormal corneal biomechanics and its significant correlation with field parameters may be harmful reasons for the generation of open-angle glaucoma with normal IOP.

Besides, Eliasy et al. [44] considered the biomechanical behavior of healthy cornea measured by Corvis ST tension measurement using the stress–strain index. The method can help optimize corneal surgery such as refractive surgery and the introduction of corneal implants. Perez-rico et al. [45] studied the influence of diabetes on Corvis ST measurement process. Comparisons were made using the ocular response analyzer and Corvis ST analysis to determine the influence of disease duration, hyperglycemia, and hemoglobin levels on these parameters. Poor glycemic control in diabetics affects corneal biomechanics as measured by the eye response analyzer and Corvis ST, which may suggest that high IOP is independent of central corneal thickness. The measurement of corneal biomechanics should be considered in clinical applications.

### Ocular tissue biomechanical analysis using finite element method

Finite element analysis software is favored by researchers because of its powerful simulation function (Table 2). A variety of complex structures can be established, the database contains the rich unit type and material properties to facilitate the establishment of the physical model. The construction of the physical model of a reusable can exert various load cases and the model can be visualized structure of internal stress and strain concentration conditions. This method can effectively avoid animal experiments and medical ethics problems. The combination of computer simulation data and clinical study results provides a possible solution for the prediction and treatment of early ocular abnormalities. Due to its advantages, researchers often apply finite element analysis to the study of mechanic-related pathogenesis and treatment of glaucoma and eye surgery.

**Table 2:** Ocular tissue biomechanical finite element analysis

Ocular tissue	Research contents	Refs
ONH	ONH cell damage	[50–56]
Lamina cribrosa (LC)	Deformation of lamina cribrosa	[57–59]
Sclera	Scleral surface displacement	[60]
Iris	Iris stiffness measurement	[61]
Pupil obstruction and aqueous outflow	Pupillary block	[62]
Trabecular meshwork	Hardness of the trabecular meshwork	[63, 64]
Cornea and lens	Corneal biomechanical deformation response	[67–73]
Other eye tissues	Finite element analysis to assist surgery	[74–82]

#### Ocular finite element modeling

Finite element (FE) modeling is generally regarded as an effective method to quantitatively analyze the pathogenesis of glaucoma. In recent years, research has focused on building local eye models. Karimi et al. [46] simulated the biomechanical stress and strain of ONH using FE method, and various mesh elements were used to investigate the effect of mesh type on the results. The 20-node hexahedron element produces the most accurate results in complex models. The calculation results of the 10-node tetrahedral element and 20-node hexahedral element are very similar and can be exchanged for a short calculation time. Linear element types do not produce acceptable results. To get closer to the real eye tissue, Zhou et al. [47] proposed a new biomechanical material model of the whole eye. Collagen content in the eye tissue measured by X-ray scattering was expressed by Zernike polynomials covering the cornea and sclera. Based on the user-defined material model, a fine mesh FE model with the specific geometry of the human eye was established. The model was then used in iterative inverse modeling studies to derive material parameters.

However, local eye tissue modeling responses are often not comprehensive enough to include global eye structure models, so more effective information may be obtained in the study of ophthalmic disease biomechanics. As increased IOP may be a major danger factor for glaucoma, Dai et al. [48] constructed a global FE eye model and simulated the effect of elevated IOP on eye structure. A refined global eye model was established using ANSYS software to study the relationship between IOP and biomechanical response. First, the pressure transfer process of elevated IOP was analyzed to simulate the effect of elevated IOP on glaucoma. Then, biomechanical responses of the anterior eye segment under different pressures were analyzed by simulating non-adhesion of the iris and posterior sclera. The model not only simulates the effects of elevated IOP on ocular structure, but also reveals the process of pressure transfer from the anterior segment to the ONH. The local mechanical properties of the eye structure obtained from the global model are consistent with the previous results. This global model may shed light on multifactorial glaucoma research. Studies have shown that eyeball design can affect the results of biomechanical analysis. Issarti et al. [49] assessed the mechanical contribution of intraocular structures to corneal deformation by establishing the whole eyeball FE mode. The corneal mechanical deformation under different mechanical conditions revealed that the lens, iris, and muscle are also the main factors that should be considered.

#### Glaucoma-related ocular tissue biomechanical finite element analysis

##### Biomechanical properties of ONH and lamina cribrosa (LC)

At present, biomechanical analysis of glaucoma tissues by finite element method is the main application type to explore the pathogenic mechanism and the application of treatment. ONH cell damage is widely recognized as the direct cause of blindness in glaucoma, but the pathway of its occurrence remains unclear.

Glaucoma is partly characterized by elevated and fluctuating IOP, which in turn loads the head of the optic nerve. In addition, tissue viscoelasticity strongly affects the mechanical response of ONH to mechanical load, but its viscoelastic mechanical properties remain unclear. To determine these properties, Safa et al. [50] made micromechanical tests and constructed a mixed model containing two phase materials of the viscoelastic solid matrix. It was proved that the viscoelastic mechanical response of ONH could be revealed by the mechanism of fluid flow and solid matrix viscoelasticity. Understanding these parameters will facilitate the establishment of in vitro research models and experiments, and further explain the pathogenesis of glaucoma under different conditions.

In recent years, ONH injury caused by other factors is also the focus of researchers. Wang et al. [51] discovered that the hardness of Bruch’s membrane-choroid complex is equal to or higher than that of other eye tissues, it may have a non-negligible influence on the ONH deformation induced by high IOP. Similarly, Feola et al. [52] explored how the anatomy and swelling of choroids affect the ONH by developing finite element models of ONH. Results showed that choroids may have a great influence on the biomechanics of ONH. In addition, Jin et al. [53] developed a FE model of a normal eye, and predicted ocular pulse amplitude, choroidal expansion could affect the biomechanics of ONH during the cardiac cycle. Ma et al. [54] investigated the correlation between IOP-induced local displacement of the ONH and the thickness of the peripapillary sclera, the results suggest that the greater backward movement of ONH relative to the surrounding tissues, the thinner peripapillary tissue, and also the LC may play a major role in preventing excessive backward displacement of ONH during acute elevated IOP.

Kim et al. [55] explored the effects of heart rate changes on the dynamic biomechanical characteristic of ocular pulse and ONH. Ocular pulse amplitude OPA, pulse volume, and ONH deformation decreased with the increase of AR heart rate, while LC became hard. The influence of changes in blood pressure/heart rate on ONH sclerosis may have important implications for the pathology of glaucoma. FE simulation was used by Shin et al. [56], results revealed that mechanical stress and strain concentrated in the ONH region by the abduction of the optic nerve sheath were much greater than that of the elevated IOP. This supports the new concept that glaucoma optic neuropathy may be caused at least in part by external traction of the optic nerve, and not just the pressure from the inside of the eye on the optic nerve.

The pore shape and size of the LC are predictors of the mechanical damage to the optic nerve tissue in glaucoma. To determine the deformation of the neural tissue in the LC pore, Voorhees et al. [57] established computational models of LC, with different nonlinear anisotropy and neural microstructures based on the tissue sections of sheep’s eyes. The microscopic structure of the LC results in localized mechanical changes in neural tissue. In addition, they further measured the significant damage to the LC nerve tissue [58]. Similarly, Karimi et al. [59] proposed FE models of three human eye posterior poles, including LC microstructures and distributed nerve tissue composed of retinal axons. These models were used to estimate the stress and strain of the LC and dispersed neural tissue under acute elevated IOP.

##### Sclera, iris, pupil obstruction, aqueous outflow system, trabecular meshwork

Schwaner et al. [60] studied the biomechanics of rat sclera by inverse FE model. The scleral surface displacement was first measured by digital image correlation. The sclera was modeled as a nonlinear material embedded with collagen fibers, and used a differential evolution algorithm to fit the model displacement to the experimental data. The material properties identified were beneficial for the study of glaucoma. Pant et al. [61] carried out an image-based reverse modeling analysis to quantitatively explore the iris properties in angle-closure glaucoma people.

The iris in glaucomatous patients was stiffer than that of healthy people. It may provide a reference for the study of the mechanism in angle-closure glaucoma. Wang et al. [62] constructed 21 eye finite element models to simulate the influence of the pupillary blocking force with various pupil diameters and iris–lens channel distance. It was shown that the influence of the iris–lens channel on pupillary blocking force is more notable than pupil diameter. It would be the main potential risk for primary angle-closure glaucoma.

It is difficult to obtain the accurate location and hydrodynamic data of aqueous outflow resistance with increased IOP. Zhang et al. [63] found that the change of trabecular meshwork permeability has the greatest influence on hydromechanical parameters of trabecular meshwork and Schlemm’s canal. By using the bidirectional fluid–structure coupling simulation method, Wang et al. [64] confirmed the hardness of the trabecular meshwork in glaucoma patients is higher, and outflow from both normal eyes and glaucoma appears to be associated with stiffness of the trabecular meshwork combining finite element modeling and ocular imaging device. It also provides a reference for further research on the factors regulating the biomechanical properties of the trabecular meshwork.

#### Finite element analysis of cornea and other eye tissues

Evaluation of corneal biomechanics is the basis for the study of ocular surgery and the accuracy of IOP measurement. The biomechanical responses of the cornea and adipose tissue in dynamic IOP tests were evaluated using the inverse finite element method and inverse model [65]. Pandolfi et al. [66] have taken into account the biological tissues with multiple physical properties and typical characteristics of the ocular environment, and custom geometric models are constructed based on the parameters of imaging and in vivo testing. The acquired patient-specific model can provide information on the post-operative shape of the cornea [67].

Montanino et al. [68] proposed a numerical model for non-contact testing that could evaluate the mechanical parameters of the human cornea. This test consists of a fast injection of air on the corneal anterior surface and concluded that the internal fluid is vitally important for simulation. The effect of different scleral stiffness on corneal biomechanical deformation response under airbag load was described by the finite element model. Nquyen et al. [69] conducted the FE study of the biomechanical effect of sclera on corneal deformation response. Inflatable deformation in a two-dimensional axisymmetric fixed FE model of the whole eye is generally considered to be entirely due to IOP and corneal characteristics. The current study shows that the harder the sclera, the greater the limitation of corneal deformation, independent of IOP. It may have important clinical application value to explain cornea response under aeration load under pathological conditions. Qin et al. [70] proposed a way to measure corneal elastic modulus based on Corvis ST results. Based on the calculated elastic modulus, the corneal apical displacement was simulated by the finite element method, and the simulated corneal apical displacement was consistent with the experimental results. Based on the relationship between force and displacement of shallow spherical shells, the method of determining corneal elastic modulus based on the Corvis test is simple and effective.

Moreover, Karimi et al. [71] studied the biomechanics of healthy cornea and corneal conus by combining clinical data, FE, and artificial neural network, and established a new biomechanics-based keratoconus eye diagnosis method. Clinical biomechanical parameters of healthy and only keratoconus could be obtained by non-contact tonometer. According to the corneal geometry, the finite element model of each cornea and the same boundary and loading conditions were applied not only to confirm the biomechanical FE parameters, but also to calculate the amount of von Mises stress at the apex of the cornea. Then clinical biomechanical data and von Mises stress were combined into the artificial neural network algorithm to distinguish healthy cornea from keratoconus based on the resulting von Mises stress. These findings not only have important implications for eye care professionals to identify keratoconus as an important clinical and surgical tool, but also provide quantitative and accurate methods for understanding the biomechanical properties of keratoconus.

Additionally, Zhang et al. [72] introduced a new way to measure the mechanical stiffness of healthy cornea and corneal cone. FE modeling performed the analysis of healthy cornea and corneal cone. Stress–strain index was proposed and evaluated in earlier studies as a parameter, and was used as a method for corneal stiffness study. SSI maps can estimate regional variations in biomechanical stiffness of the cornea surface. Rahmati et al. [73] used coupled FE optimization algorithm to estimate the viscoelastic properties of healthy corneas and Keratoconus corneas. This algorithm is a non-invasive technique that can accurately estimate the viscoelastic cornea properties.

Corneal surgery mechanical impact study has established the 3D FE human eye model to verify the laser in situ corneal grinding with surgery (LASIK) on corneal biomechanics behavior, Bao et al. [74] found that when considering LASIK surgery of corneal biomechanical behavior, improving the match of clinical measurements and LASIK predictions. This result has important implications for the development of planning tools for refractive surgery. Mohamed et al. [75] developed a new numerical simulation method for corneal transplantation to study the stress and deformation of corneal tissue and donor transplantation during corneal endothelial transplantation. The results clearly showed that bubble pressure is vitally important for the stress and strain of the cornea and the stiffness and thickness of the corneal membrane.

In arcuate keratectomy, Truffer et al. [76] proposed an arcuate keratotomy scheme for personalized FE simulation. Virtual surgery was performed on patients, and a numerical model was used to optimize the surgical parameters of arcuate keratotomy, which could improve the reliability of postoperative astigmatism and reduce the risk of overcorrection. Knaus et al. [77] investigated the effect of ciliary muscle contraction on lens adjustment by using a 3D FE model. The results showed that the ciliary muscle section had a synergistic effect: the circular section contributed the most to the increase of lens thickness, while the longitudinal and radial sections did the opposite. Conversely, the function of the longitudinal and radial parts is to shift the lens forward, as opposed to the circular part. The finite element model demonstrates the complex interaction of ciliary muscle three segments during lens deformation and transformation during adjustment.

In addition, due to car accidents, earthquakes, gang fights, and other reasons, glass fragments can collide with the eye, causing numerous scars, which can permanently affect vision. Complications from collisions with the eye and subsequent damage to various parts of the eye can be difficult to diagnose. Karimi et al. [78] constructed a 3D human eye model to evaluate the impact of glass fragments on human eyes. As the speed of glass fragments increases, the stress in the center of the vestibule, like the cornea, water body, and iris, increases. But for parts located outside/behind the eye, especially the optic nerve, a reduction in pressure is observed by increasing speed, with little damage to the optic nerve. These findings not only help to understand the stress/tension in the eye at different speeds, but also help to provide ophthalmologists with initial information for better diagnosis after eye injuries from fragment-like (small object impact) injuries.

To develop a detailed human orbital FE model and verify it by analyzing its behavior under blunt trauma pressure. Foletti et al. [79] used a modified 3D FE human head, which is the most complex model developed to date, to advance the understanding of the mechanics of optic nerve injury. Singman et al. [80] developed a model of the head with a biofiber-mesh eye socket. This study represented the first published biological neural simulation using the full length of the optic nerve, in which the eye socket is integrated with the entire head. Song et al. [81] used a 3D FE model to study the orbital bone development of congenital microphthalmia. Periocular biomechanics mainly focuses on the medial center of the orbital wall and gradually extends to the entire orbital wall. Deck et al. [82] study the FE eye model of retinal hemorrhage in shaken baby syndrome. In the retina, the slightest tremor causes four times as much pressure as the most severe shock, and shaking a baby causes extreme eye strain compared to falling.

### Microfluidic method in ocular chip

Numerical simulation has been recognized as an effective means of eye disease biomechanical research, but for most studies, the eye model is often simplified, and biomechanics analysis was conducted under ideal conditions without considering other factors, which may lead to the results deviation of the numerical analysis and the actual situation [107]. In recent years, researchers have shown great interest in alternatives to in vitro models of the eye (Table 3), allowing for the addition of dynamic fluid flows to better mimic the physiological structure of the eye [108, 109].

**Table 3:** In vitro biomechanical study methods of eye tissue

In vitro methods	Research object and classification of biomechanics		Refs
Ophthalmic professional medical equipment	Corvis ST, Springback tonometer, Goldmann tonometers, ocular response analyzer		[40–45]
Finite element method	Finite element modeling	Global modeling and local modeling	[47–49]
	Glaucoma-related	Optic nerve head, lamina cribrosa	[15–17, 50–59]
		The sclera, iris, pupil obstruction, aqueous outflow system, trabecular meshwork	[60–64]
	Cornea, lens, and other eye tissues	Corneal surgery mechanics	[66–75]
		Eyeball injury	[77–82]
Microfluidic chip	Platform	Eye pressure sensor, tear component sensor, eye chip	[83–96]
	Drug preparation and delivery	Microfluidic eyeball microfluidic device for nano-drug preparation and drug delivery	[97–106]

Organ microfluidic chip is a transformative technology that can reproduce the human organs in vitro disease model [110]. The development of these technologies will increase our understanding of the different structure of eyes of basic physiology, enables us to examine the unknown aspects of eye disease pathogenesis, and as a substitute for clinically relevant to assess eye treatment [111].

In addition, since tears include many biomarkers, contact lens sensors can be used to directly measure different parameters such as glucose, urea, protein concentrations, ions [112], IOP, and corneal temperature, noninvasively. Microelectromechanical machining technology [113] enables contact lenses with detection electrodes and tiny structures to be used as wearable devices for biomarkers monitoring and delivery of drugs for treating eye diseases [114]. The combination of eyeball physiological information monitoring and drug delivery system will become the trend of personalized ophthalmic disease diagnosis and treatment [115].

#### Ophthalmic disease research platform based on microfluidic chip

**Fig. 1:**
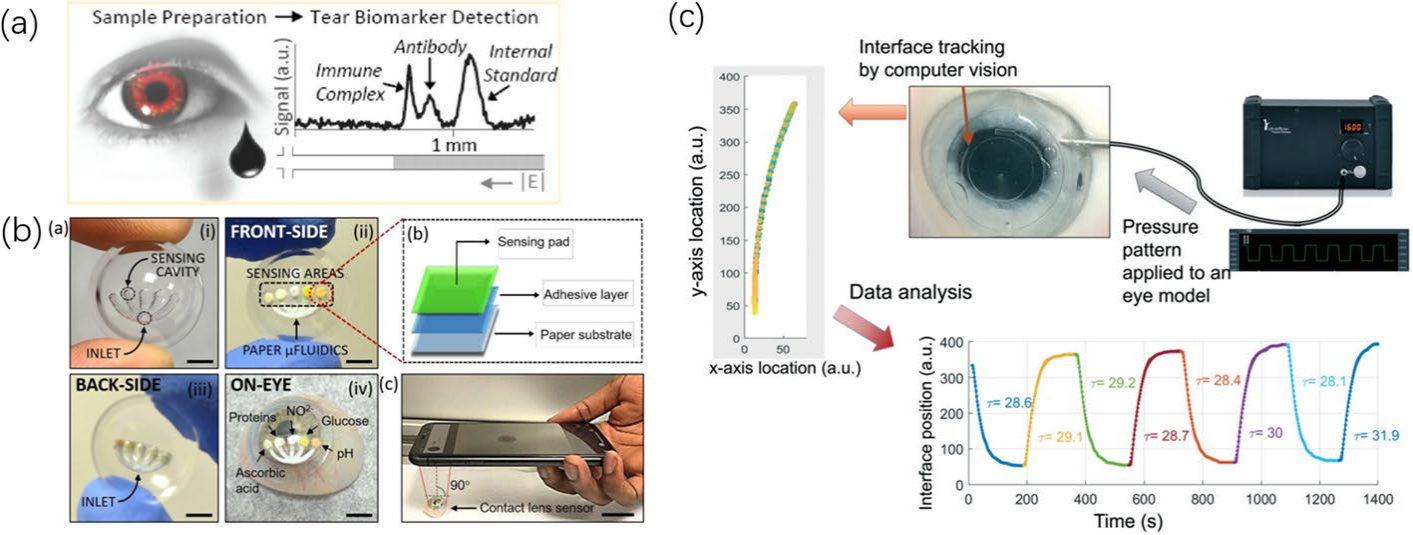
**a** Microfluidic tear component analysis platform proposed by Karns et al. [83] Reproduced with permission from Ref. [83]. Copyright 2011, American Chemical Society. **b** Colorimetric μPad device for tear electrolyte analysis proposed by Yetisen et al. [87]. Reproduced with permission from Ref. [87]. Copyright 2020, Royal Society of Chemistry. **c** Wearable intraocular pressure sensor and detection system based on contact lens proposed by Araci et al. [90] Reproduced with permission from Ref. [90]. Copyright 2019, Royal Society of Chemistry

Biomarkers contained in tears provide an important reference for the non-invasive understanding of physiological disease processes. Karns et al. [83] developed a microfluidic homogeneous immunoassay chip for a rapid, quantitative and specific measurement of endogenous tear protein (lactoferrin) biomarkers in human tears. The accuracy of the immunoassay chip was within 15% of ELISA, and the detection limit was 3 ± 2 nM (Fig. 1a). Sonobe et al. [84] proposed a novel paper-based ELISA analytical device for assessing tear lactoferrin in dry eye people. Results showed that lactoferritin concentration was correlated with Schirmer test values and tear film rupture time, but negatively correlated with ocular surface disease index, luciferin, and rose Bengal scores.

Microfluidic fluorescence analyzer has a broad application prospect as a preclinical and clinical diagnostic instrument. Commercial fluorescence microscopes, however, are expensive and time-consuming. It is not conducive to the portable and wearable applications of microfluidic chips. Pestana et al. [85] developed a dedicated low-cost fluorescent microfluidic device reader for reading interlayer immunofluorescence analysis equipment and for detecting vascular endothelial growth factor ligand concentrations in eye fluid samples. In addition, the detection of tears facilitates early diagnosis of eye diseases, and monitoring of high-risk subjects. Yetisen et al. [86] designed a μPad system for tear electrolyte analysis (Na^+^, K^+^, Ca^2+^, and pH), including a sample collection capillary, a sample dilution reservoir, and a μPad for electrolyte analysis, and its fluorescence output was measured using a smartphone readout device.

Changes in the composition of tears are signals of ocular and systemic metabolic processes, so they can be utilized to assess physical health. Moreddu et al. [87] proposed paper microfluidics integrated into laser-implanted commercial contact lenses for detection of biomarkers. In vitro measurements include the colorimetric way, which collects, stores, and analyzes readings using a customized tear diagnostic smartphone app prototype (Fig. 1b). The application demonstrates the device’s potential for discrete measurements during medical diagnosis. In addition, discovery further developed a laser-cut wearable contact lens sensor for the analytes monitoring in tears [88]. Microfluidic systems are implanted in commercial contact lenses via CO_2_ laser ablation. The microchannel consists of a central ring consisting of four branches, and the biosensor is embedded in a microcavity at the end of the branch. Colorimetric readout based on the nearest neighbor model is carried out by MatLab algorithm of smart phone. They then further developed a laboratory platform for contact lenses using multiaxial femtosecond laser ablation [89] to quickly and accurately etch microfluidic networks on the surface of contact lenses. Production of functional microfluidics components such as flow valves, resistors, multi-entry geometry, and dispensers were realized with custom seven-axis femtosecond laser systems.

The measurement of IOP is also an important means of detecting glaucoma. The expansion strain sensor, which works by detecting volume changes in microfluidic channels, is highly sensitive to biaxial strain and is made only from soft and transparent materials, making it easy to integrate with smartphones. These characteristics have advantages for contact lens-based IOP sensing applications. Because in the measurement of IOP, the noise generated by eye movement and pulsation will affect the measurement of IOP signal, and the use of electronic components for filtering will bring inconvenience to the human body. The microfluidic equivalent circuit can realize analog/digital conversion, filtering, and other logical operations through flow channel design and fluid parameter modification. Araci et al. [90] adopted the microfluidic equivalent circuit to achieve low stability and noise suppression of wearable microfluidic sensors and improve the signal–noise ratio of ophthalmic applications. Suppression of noise caused by eye pulsation, blinking, etc., without the need for electronic components was realized (Fig. 1c).

In addition, the study of biomechanics and pharmacokinetics of eyeballs by constructing in vitro eyeball model and using the microfluidic method is also one of the research hotspots in recent years. Beissner et al. [91] applied a previously designed dynamic microtissue engineering system with a pre-validated human corneal structure to obtain an improved test platform. The platform offers a huge opportunity to improve common in vitro drug testing procedures.

**Fig. 2:**
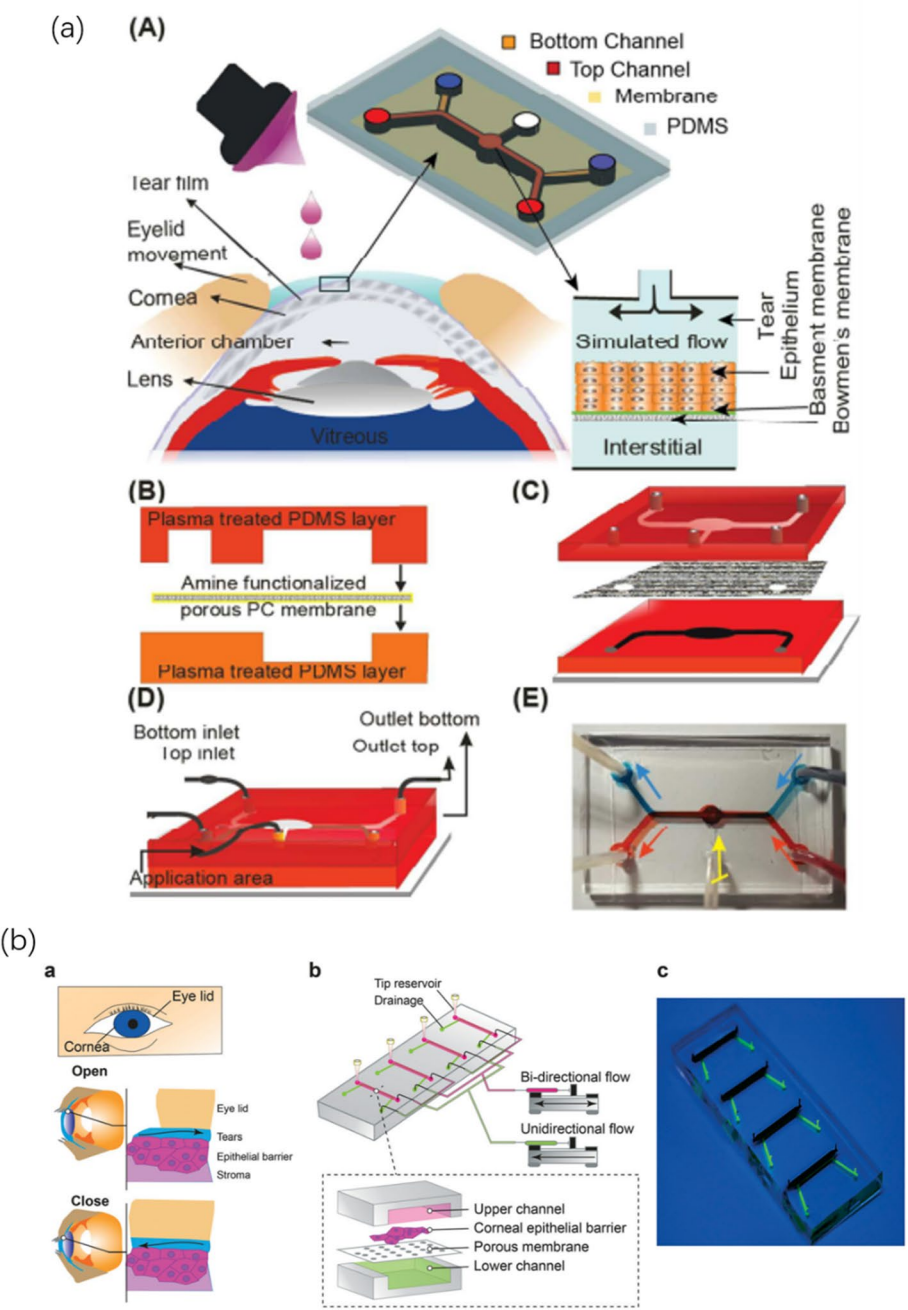
**a** An in vitro eyeball model platform developed by a microfluidic eyeball cell chip was used to study pharmacokinetics proposed by Bennet et al. [92]. Reproduced with permission from Ref. [92]. Copyright 2018, Royal Society of Chemistry. **b** The human corneal barrier and blink reconstruction platform based on the microfluidic chip is used to study the development of ophthalmic drugs proposed by Abdalkader et al. [95] Reproduced with permission from Ref. [95]. Copyright 2020, Royal Society of Chemistry

The cost and ethical criticism of ocular drug development of animal pharmacokinetic studies made it necessary to develop an in vitro model to study corneal. Bennet et al. [92] prepared a porous membrane embedded in the microfluidic platform to separate the chip into top and base sides. Immortalized human corneal epithelial cells were grown on the membrane to create a microengineered corneal epithelial chip (Corneal chip), a model that can imitate the environment of the human cornea. The current model in the further study of angiogenesis in vitro lack of extensibility (Fig. 2a). Ko et al. [93] developed a plastic-based microfluidic chip for in vitro reconstruction of the three-dimensional vascular network, and carried out the human eye angiogenesis model on the injection molding microfluidic chip. The chip provides a simple fluid mode for constructing cell culture microenvironments. This model can be used not only in normal and pathological vascular studies, but also in basic studies of ocular neovascularization.

Isopropanol silicone oil is commonly used as a tamponade to treat complex retinal detachment and proliferative vitreoretinopathy, which is prone to emulsification in vivo and may cause inflammation. Lu et al. [94] used a microfluidic device and an in vitro “eye-on-a-chip” to evaluate whether the addition of polymer silicone oil could facilitate the injection and removal of silicone oil. The results show that the presence of polymer silicone oil can increase the tensile viscosity and prevent the decomposition of silicone oil from the matrix to form emulsion droplets.

Human corneal epithelium coexists with tear fluid and exhibits its barrier function under dynamic blinking conditions. However, the current in vitro culture environment of corneal epithelial cells lacks dynamic flow conditions to reproduce the shearing stress during blinking. Abdalkader et al. [95] developed a microfluidic platform that can dynamically cultivate the human corneal barrier by recreating blinking. It is expected that the multi-corneal barrier device on a chip will open new possibilities for the development of ophthalmic drugs and will contribute to the study of the effects of blink shear stress on the ocular surface (Fig. 2b). Bai et al. [96] conducted the preparation of a novel 3D chip cornea using primary mouse corneal epithelial and endothelial cells. The separation and culture scheme of primary corneal epithelial cells and endothelial cells was used to construct a microfluidic 3D microengineered cornea based on primary cells. This chip overcomes the shortcomings of 2D cell culture and realizes corneal function and delivery of drugs.

#### Preparation of ophthalmic drug and drug delivery device based on the microfluidic chip

For ophthalmic diseases, surgical implants and implanted drugs are currently the main treatment mode. However, from the perspective of patient comfort (such as regular insertion and removal of implants), they are not accepted by users. Currently, the main non-invasive treatment options, such as eye drops, are only 1–3% of the drug can reach the tissues in the eye. Improving the controlled release of ophthalmic drugs is a key challenge in addressing the local administration of hormones and drugs.

Polymer particles are capable of drug loading and controlled release, and their biocompatibility and biodegradability make them powerful tools for non-invasive drugs. The glaucoma drug betaprolol, for example, is released more slowly and for longer when combined with polymer ion-exchange resin particles suspended in an adhesive medium. However, the preparation of uniform particle size, controllable particle size, and high drug loading, good degradation of polymer particles is the main problem. The preparation of polymer particles based on a microfluidic chip provides the possibility for the preparation of polymer particles. Polylactic acid–glycolic acid copolymer (PLGA) is one of the most widely used biodegradable materials [116]. Leon et al. [97] used microfluidic technique to prepare multidrug polymer particles for the treatment of external glaucoma. They manufactured multidrug-loaded biodegradable polymer particles by loading Latanoprost and dexamethasone onto monodisperse microparticles (approximately 150 μm in diameter) of biodegradable PLGA, the monodisperse oil-in-water emulsion was generated using a capillary microfluidic device, which was then evaporated by the thin film to produce monodisperse particles with a diameter of = 150 um and a standard deviation of  < 5%. The parameters of drug loading, drug release, in vitro adhesion, and the measurement of local drug use were verified and optimized. The study is expected to reduce the number of times patients receive eye drops and improve the effectiveness of drug delivery.

In addition to polymer micron particles, drug nanosuspensions were found to have high bioavailability. Drug nanosuspensions are composed of micron drug particles suspended in a dispersed medium and stabilized by polymers or surfactants. The current preparation of nanosuspension drugs is mainly by grinding large drug particles or by precipitation of drug molecules to construct nanodrugs, and the main development direction is the latter. Ali et al. [98] compared the preparation of ophthalmic hydrocortisone nanosuspension by the microfluidic nanoprecipitation method and wet grinding method. Hydrocortisone is a widely used steroid to treat all kinds of eye inflammation. The particle size, shape, and crystallinity of the suspensions prepared by the two methods were characterized. The results showed that hydrocortisone nanosuspension was developed by the microfluidic nanoprecipitation method. As a novel, simple and economical drug nanocrystalization technology, the drug action time of nanometer suspensions could be significantly prolonged.

So far, researchers have developed many eyes organ in vitro models, from 2D cell culture models based on single-cell culture, cell culture models, or trained models, to the three-dimensional organs, 3D printing chip systems, and organs, organs including local and overall, in vitro drug screening and disease research contribution. In particular, the combination of microfluidic technology can make the in vitro study of drug delivery effects very useful [99]. Micro-drug delivery devices are also vitally important in the controlled release of drugs. Precision ophthalmic drug delivery devices combine mechanical, electronic, and microfluidic functions [100].

Since most ophthalmic drugs were delivered via topical eye drops, there is a huge demand for the controlled release of drugs from ocular biomaterials. Kaczmarek et al. [101] constructed a long-term wear-resistant microfluid hydrogel contact lens by adjusting the composition of biomaterials to realize the controlled release of dexamethasone over 60 days. Phan et al. [102] explored a new in vitro eye model for fluconazole release from a variety of commercial contact lenses. The eye model is prepared by 3D printing and filled with PDMS, which can obtain a cheap model of the eye and eyelid. Use injection pump as a fluid driving force. Compared with 3D printed eye models, the vials had higher drug release. The drug is quickly released from the contact lens within the first 2 h, followed by a plateau. Rapid drug release can be achieved by using vials as a drug release system. The volume of tears significantly speeds up the drug release process. Subsequently, they further refined the model [103] of the eyeball and eyelid masses to simulate physiological tear volume. The integration of human or animal corneas or human corneas will allow for more complex in vitro eye studies. Combined with the injection pump, the platform was constructed for studies to evaluate drug delivery and deposition in contact lenses.

Similarly, because most used ophthalmology in vitro drug release study is carried out under static conditions, and are not fully considering the influence of flow dynamics, need to take into account the tear volume and flow in the body, Pimenta et al. [104] designed a microfluidic unit to simulate the tear continuous, the volumetric flow rate, and its small volume. The release kinetics of a drug system containing diclofenac was compared with that under static condition by using the microfluidic chip, and the results showed that the release kinetics under dynamic condition was slower. Subsequently, they investigated [105] plasma-assisted grafting of 2-acrylamide-2-methylpropane sulfonic acid or 2-(methylacryloxy) ethyl dimethyl-(3-sulfopropyl ammonium hydroxide) to the surface modification of hydrophilic acrylic materials for the preparation of intraocular lenses. Its load of the endophthalmitis antibiotic moxifloxacin was shown to significantly prolong the drug release time (up to 12 days) in vitro. Silva et al. [106] used moxifloxacin hydrochloride imprinted silicon-based hydrogel as a contact lens material for prolonged drug release. Contact lenses can be used as a platform for drug release for the treatment of eye infections, but their typical release period still needs to be extended. The microfluidic unit was used to simulate the flow of ocular surface fluid. The results showed that the release period of *Staphylococcus aureus* and *Staphylococcus epidermidis* was about 2 weeks.

## Conclusion and future trend

Ocular diseases are closely related to the physiological changes in the eyeball and its contents. Using biomechanical methods to explore the relationship between the structure and function of eye tissue at multiple scales is helpful to reveal the pathological processes. Therefore, this text summarized the biomechanical research status of several ophthalmic diseases (including glaucoma, high myopia, and diabetic eye disease). The biomechanical research of eyeballs based on computer modeling is the mainstream at present, but the simplified model and ideal conditions may result in different conclusions from the real situation. The combination with ophthalmic medical equipment may be helpful to the diagnosis and surgical treatment of eye diseases. With the development of microfluidic organ chips and wearable microfluidic chips, more and more refined biological models of the eyeball can be realized through 3D cell culture, which undoubtedly plays a good supplement to the in vitro biomechanical research of the eyeball. In addition, the preparation of eyeball nanomedicine based on microfluidic chips, wearable intraocular metabolite sensors, and eyeball drug controlled-release microfluidic devices provide new ideas for the treatment of systemic ophthalmic diseases. It is believed that more theoretical biomechanical models of eyeball diseases will be combined with in vitro physiological models in the subsequent studies to promote the progress of ophthalmic medicine.

## Data Availability

Not applicable.

## Abbreviations

IOP: Intraocular pressure
ONH: Optic nerve head
LC: Lamina cribrosa
RGC: Retinal ganglion cells
LOX: Lysine oxidase
SMILE: Small incision lenticule extraction
LASEK: Laser-assisted subepithelial keratoplasty
ORA: Ocular response analyzer
GAT: Goldman applanation tonometer
FE: Finite element
PLGA: Polylactic acid–glycolic acid copolymer
